# Lecture upon Lycopodium

**Published:** 1888-11

**Authors:** J. T. Kent


### LECTURE UPON LYCOPODIUM.

FROM NOTES OF LECTURE BY PROFESSOR J. T. KENT.

(Continued from page 468.)

The fan-like motion of the alæ nasi is said to be a keynote to Lyc. Now, how and when will it occur? Suppose a case of pneumonia in a child, or a low form of bronchitis; there comes on an attack of violent dyspnœa. You will then see the dilated nostril, with wrinkled face; the flapping of the wings back and forth; heaving of the chest; heaving of the abdomen; more or less sweating; perhaps coldness and blueness of the surface. Here, then, this symptom stands out prominently; this, then, is pre-eminently a Lyc. picture. If the patient be a child, examine the diaper for red sand or turbid urine; see if the urine be reddish, or if there be a brick-colored deposit; also see that there be no opposing symptom.

Copper-colored eruptions, such as are found in syphilis, are cured by Lyc. Copper-colored and brown liver spots upon the forehead, upon the face, upon the abdomen and chest, anywhere upon the body.

Lycopodium is a great liver remedy, producing portal congestion, duodenal catarrh, catarrh of the stomach and bowels, all the peculiar features of jaundice, liver patches, melancholia, weakness of mind, almost amounting to imbecility. It has a peculiar silliness, a childish look; it seems to be a lack of understanding of what is said and of what is going on; the mind is enfeebled; the patient will stand and look at you, smile, laugh, and smirk, and not in the least comprehend what you are saying. The condition of the mind is clearly expressed on the face.

The face is sometimes pallid and sometimes flushed. There are flashes of heat, like Lach., Sep., Sulph.; often flashes of heat over the entire body. Hot and cold spots alternate in the back; burning like coals of fire between the scapulæ.

Feet are cold and damp, and this condition extends up the extremities to the knees; cold as ice; much cold sweat; cold night-sweats, with prostration and nervous weakness.

The tongue is a great index to the condition of the stomach and fluids of the body, and has all the symptoms from dryness and redness to the black and cracked conditions found in zymosis.

We find in Lyc. the oscillation and darting to and fro of the tongue, the trembling, heaviness, and stiffness of the tongue, causing indistinct speech.

Lyc. has all kinds of bad tastes and foul breath. In a general way offensiveness is a characteristic of the drug; the perspiration, the stool, the flatus, the eructations, all the excretions of the body, are offensive. So we find it in the mouth and breath, with slimy, heavily coated tongue, or, as in case of zymosis, covered with a dark, nasty coat; sordes on the teeth; exudations of blood, like Phos., Chin., Zinc, Rhus, and many others.

In the characteristic sore throat of Lyc., diphtheria, tonsillitis, pharyngitis, suppurative or ulcerative throats, both the pain and soreness begin upon the right side, progressing to the left; the throat is much less sensitive than the amount of inflammation would lead you to expect; is *agg.* by cold drinks; is *amel.* by warm drinks, and in which conditions it is directly opposed to *Lach.*, which begins on the left side, progresses to the right; is greatly more sensitive than the amount of inflammation would lead you to expect; is greatly *agg.* by warmth, by warm drinks, and has great *agg.* from covering or the slightest pressure about the neck. In a sore throat beginning upon the right side, proceeding to the left, with absence of leading characteristics, you will find Lyc. stands pre-eminently at the head of the list.

Lyc. is peculiar in its appetites and desires; it desires sweets; oysters, and he gets sick from eating them. May be cured by Lyc. Coffee makes him sick. You will find, as a rule, where there is a great amount of portal congestion and inflammation of the liver, there is an aversion to coffee; at first it seems to agree, but he soon learns that even the taste of coffee makes him sick, that coffee *agg.* all conditions, a few mouthfuls causing satiety.

Among the stomach symptoms, sour vomiting is quite characteristic. The stomach symptoms are temporarily relieved by hot drinks; cold and cold drinks make them worse; the patient may even vomit cold water; hot water, as hot as he can drink it, as hot as mouth and throat can tolerate it, is comforting, and *amel.* many of the symptoms. Hunger, but a few mouthfuls cause a feeling of satiety. Sour vomiting, sour eructations, sour gases; in fact, a very foul stomach; great nausea, general weakness with vomiting of food and bile.

*Lyc.* will be found one of the most useful remedies in corroding ulcers of the stomach. Don't think of Lyc. for a corroding ulcer or ulcer of the stomach, or for a thickening of the pyloric orifice; don't think of it given as a specific in that disease, but that in these manifestations you will frequently find Lyc. symptoms, and will find, as a result of proper selection and application, Lyc. to be curative.

The old masters regarded red sand in the urine as a strong characteristic of this remedy, although many other remedies have red sand in the urine. In connection with the low condition of the body, the debility and prostration of body and mind, you can hardly be misguided in selection.

In the region of the liver we have sensitiveness to contact, soreness and aching as from a shock, tension and drawing.

One foot cold and another hot is wonderfully characteristic of Lyc. when not associated with paralysis. In the latter case it is, of course, due to the imperfect circulation.

Tension in the hypochondrium, followed by gall-stone colic. Compare Berb., Bell. The usual practice is to give Chloroform to relieve a patient in gall-stone colic. A homœopath has a better method and surer relief in this state, even if it is called mechanical. A stone that is small enough to enter the orifice of the duct can pass through, as the duct is of equal size throughout, and except there was spasmodic contraction of the coats from irritation by the passage of a roughened or hardened substance, there could be no gall-stone colic. I have stopped these pains in a few minutes with Belladonna, also Berberis has cured them.

In a patient suffering for years in this condition, passing gall-stones constantly and with great suffering, Berb-v. has effected a permanent cure. What became of the gall-stones? I don't know. Relief is the first thing you should cudgel your brains to procure.

"Tension as of a cord." This is a peculiar symptom. Sensation as of a tight band is a symptom often felt in spinal disease. Cact-g. has the symptom; Ars. has it; Lyc. and other remedies have it; it is also quite characteristic of Alumina; a sensation as if a tight belt or cord were around the body; it is also very characteristic of Arg-nit.

The complaints of the stomach and abdomen are characterized by great flatulency; the abdomen feels as if it would burst from flatulency. (Compare Chin., Carb-veg.) Sensation as of something moving up and down in stomach and bowels; spasmodic contractions of the abdomen; colicky pains. Duodenal catarrh, resulting in bilious colic, that is about what it amounts to. *Lyc.* may not always be the remedy, but it produces the most typical form of duodenal catarrh, hence, jaundice, liver disturbances, yellowness of the skin, and brown spots. In dropsies dependent upon some disturbance of the liver and portal circulation think of *Lyc.*; in peritonitis consequent upon hepatitis.

Great accumulations of flatus, which becomes incarcerated, pressing outward with a feeling of distention; again pressing downward on the rectum and bladder, with great fermentation in the abdomen; brown spots on the abdomen.

In congenital hernia, Nux-v. and Lyc. are most frequently indicated, and completely curative when so indicated. Congenital hernia of the right side is most likely to need Lyc., while that of the left side most frequently needs Nux.

Renal colic, right ureter to the bladder, with stinging, tearing, digging pain, as if a calculus of small size were tearing its way through to the bladder. Berb. or Lyc. will probably be the best remedies. In Berb. the pain radiates from the kidney; pain may be either side, may be below the kidney, but is sure to be a radiating pain.

Lyc. produces great weakness of the rectum—paralytic—which is a loss of power, of force, and of sensation. It has also characteristically ineffectual urging; straining without effect, due, perhaps, to the weakness, which allows straining only of mild character. Lyc. is a grand remedy in constipation. The patient will go several days without desire for stool; the rectum fills; the urging comes with the pressure of the accumulated fæces, with effort at expulsion and only a partial emptying of the rectum, leaving a feeling of fullness and uneasiness, caused by the remaining fæces, and classing it with Sep. Sep. has the sensation of lump in the rectum after stool, caused by the remaining fæces.

Alumina has the filling of the rectum, with inability to expel the fæces, but it is characteristically a soft stool, with no urging and no desire for many days, and then difficult expulsion.

Nat-m. has constipation for many days; accumulation of fæces; ineffectual urging; great straining without desire; with the sense of fullness and uneasiness after stool.

Silicea has this debilitated state of the rectum—no ability to expel the stool, which is hard as knots, very large, packed in a mass, and requires a great amount of straining; in Nat-m. and Lyc. there seems to be a constriction at the anus preventing the expulsion. In Sil., after protracted effort, even after becoming quite exhausted, he finally gives up in despair and the stool slips back into place. No other remedy has that symptom of slipping back recorded of it as pathogenetic, although Alum. and Nat-m. have cured when these symptoms were present, they are so closely allied. Perhaps Nat-m. is strongest in the sensation that something held, or that a band prevented the passage of fæces.

Verat. and Con. have constipation with large, hard stool, ineffectual straining and urging; so have other remedies, but these mentioned are very characteristic of great constipation with damming up of the rectum.

I should also mention Nux-v. and Nux-m. as having this state. Nux-v. is opposite, having constant and ineffectual urging, with scanty stools, expelling but a little lump at each effort. Nux-m. has this state going on for days, no desire, no urging, the rectum becomes filled and seems entirely paralyzed.

The conditions belonging to these remedies, so nearly alike, are so hard to describe, and so hard to distinguish the one from the other in their especial sphere, that I despair of making you understand, and you must carefully compare other symptoms.

Opium has this state with no desire for stool, lasting days, but Op. has something quite guiding; a few round, hard, black balls are characteristic of this remedy. Round, hard, gray balls, Nux-v.; Alum., the stool will probably be soft, with great effort and loss of power.

Graphites should be mentioned in this connection, and has a stool of flattened balls, seeming to have agglomerated and pressed together, giving them this shape. The rectum is full; there is no urging, and there is no effort with the evacuation; the stool is covered with thick, tough mucus that looks like cooked white of egg, boiled albumen, tallowy mucus. That is *Graph.*

These remedies resemble Lyc. in this sphere of constipation, and are closely related to each other. It is often impossible to prescribe upon local symptoms alone, but the general conditions and concomitants help us out.

These remedies produce hemorrhoids, especially Lyc., Nux-v., Sep.; painfully protruding varices; enormous tumors protruding at stool, requiring to be pressed back. In hemorrhoids always study Sulph., Nux-v., Aloes, Æscul., and Lyc. You will find them cover many cases. Lyc. has discharge of blood from the rectum, with hemorrhoidal tumors; it has bleeding hemorrhoids—compare with Sulph., Nux-v.; there is a great amount of itching, crawling, and tingling in these tumors; itching of the anus, with burning, Sulph.; burning, Ars.; tumors blue, burning upon lightest touch, Mur-ac.

Lyc. has a wonderful sphere in the urinary organs and their excretions; great aching in the kidneys, relieved by passing urine; herein we get the symptoms expressed by pain in the back, relieved by passing urine; great pain and distention of the bladder, relieved by passing urine; cutting and burning before and during urination is quite characteristic. Cutting, sticking pains, down the ureter to the bladder, a pain that you might imagine to be caused by the passage of a small calculus along the ureter; it lasts for a few hours or a few minutes, and is followed by burning and smarting, particularly in the right groin. Generally Lyc. will cover the case. The paralytic weakness found in the rectum we will also find in the bladder; weakness with lack of power to expel the urine; he must wait a long time to expel the urine, wait and press. A number of remedies have that symptom, and as many of them are not found in the repertories, I will give you the list: "Must wait a long time for the urine to pass"—Alum., Apis, Arn., Cann-i., Hep., Lyc., Mur-ac., Nat-m., Rhus, Raph., Secale, Sep., Sil., Stram., Taxus-b. Another symptom closely allied to the one just given is this: "Must *press* a long time to pass the urine;" wait and press. For this we have Apis, Cact., Hep., Prunus-sp., Raph., Secale, Stram., Taxus. Again, a symptom somewhat similar is this: Can only pass urine while at stool—Alum., Amm-m., Laur., Nat-ph., Selen. Stramonium has this peculiar symptom: Must continue to press; if he pauses to take breath the urine ceases to flow, and he must again press. It is characteristic of Lyc. that the child screams before passing water; just about as the water begins to flow it screams, but *is relieved* by the flow. Close observation will probably show you red sand in utensil or in the diaper. Sarsap. child cries before and during urination; cries while flow continues, and you may find on the napkin pale or grayish sand. Sarsaparilla is a great remedy for these little calculi. In Lyc. the urine is turbid, as if containing bile; sometimes like old cider; sometimes small blood clots, red flocculi, and bloody urine; urine is high-colored and deposits red sand; bearing down with frequent desire to urinate; pain worse lying down, especially at night.

Incontinence of urine, important especially if there is red sand; suppression of urine—no urine secreted, as in low forms of zymosis and nervous affections; head affections; congestions to the brain; neglected pneumonia; acute disease of some days' standing.

A sensualist, disturbed all his life by excessive sexualities; masturbation; great prostration; milky urine, like Ph-ac. In women who are pregnant or are distressed by uterine affections, exhibiting the symptoms of milky urine, you would often find symptoms of Sepia. Bloody urine, *agg.* by chronic catarrh, is very characteristic of *Lyc.*, but you must learn to distinguish it from many other remedies. Canth. has bloody urine, with great burning and tearing. There is the pressing in Lyc. and a passive flow of blood, seemingly from the cutting of the calculi in their passage. Burning after urination is found in several remedies. Canth. has burning *before*, *during*, and *after*. In gonorrhœa, with an irritable bladder, burning and smarting *after* micturition, lasting some time, you should think of Natr-m.

In relation to the male sexual organs we have strongly marked impotency, relaxation, and sexual weakness. This condition is most likely to be found in those who have abandoned themselves to a long life of sexual indulgence, who, at last, try to reform, to break off their habits. Becoming tired of the life, the drinking, the carousals, and sexuality, he becomes exhausted. As soon as he tries to sober up, to be more manly, he finds himself impotent, weak, with relaxation of the organs. Desire may still be strong, but he is unable to obtain an erection; during an embrace falls asleep; this is strongly Lyc. The text reads impotency, penis small, cold, and relaxed. Same conditions may arise from poisoning by Chlorine, or from onanism; short-lasting erections, too short to enable completion of the act. Widowers suffering greatly from unrequited sexual passion—(Con.) Widowers, stopping short in the marital relations, losing their power, accompanied by excessive and exhausting pollutions, involuntary seminal emissions, will usually be cured by Sulph., Lyc., Calc.

A state closely allied to this, and yet quite opposite, is where the sexual instincts are too active; there is most intense sexual desire; erections night and day, in which you will find Picric-ac., Plat., Con. very useful. If there are tumors behind the corona, think of Thuja. Lyc. is a most excellent remedy in a gonorrhœa, with a yellowish-green discharge, resulting in impotency.

Lyc. has produced and cured nymphomania, with a terrible teasing desire in the external organs. Discharge of wind from the vagina, accumulation of wind in the vagina, Brom., Lyc., Nux-v., Sang., Lac-can.

Lyc. has great dryness in the vagina, also great burning in the vagina during coition (Kreos. and Sulph.), sometimes so great as to prevent coition. Aversion to coition and to the opposite sex. Bell., Ferr., Lyc., Nat-m., and Sep. all have extreme dryness of the vagina with painful coition.

Lyc. has a very troublesome, thick, yellow leucorrhœa, likely to cause burning. In Kreos. burning seems to come from acrid discharges, causing the irritation. Sulph. has burning in vagina during and after coition and at all times—more marked at coition; it comes and goes. Ars., Bell., Bry., Con., Tarent. have burning almost as if hot coals were in the vagina, sometimes so severe as to prevent sleep and cause her to walk the floor. Tarent. has burning as of fire in the uterus with tenderness of the uterus. Sensitiveness to touch. Tereb. has great burning in the uterus. Another remedy, having great burning in the uterus, which, according to some, should be mentioned with caution, is Anthracinum, prepared from the poison of the Anthrax. Two remedies have for characteristics burning in the region of the cervix, *agg.* by touch. This would be discovered by digital examination, the patient exclaiming, "That is where the burning is;" the remedies are Sep., Kreos. A differential symptom in Kreos. would be, that during the inter-menstrual period coition causes burning and is often followed by a discharge of blood. There are other classifications and peculiarities in connection with this symptom of burning in the uterus. Burning in the uterus before menstruation, and only before, is Bufo., Carb-an., Con., and Nat-m. Burning, beginning when the flow has stopped, is especially characteristic of Canth. and Kreos.

Remedies known in relation to the burning and stinging of the mammary glands are Apis, Carb-an., Lyc., Phos., Lauro. Lyc. is prominent in the symptom of milk in the breasts of young girls, milk in the breasts when it should not be there—milk in the breasts of boys. These cases will be covered by Lyc., Puls., Phos., Cycl.; are sometimes present at puberty or at first menstrual flow. There may be hard nodules—hard nodular glands with milk in the breasts. Flow delayed until sixteen or seventeen years of age. It is a rare condition, but when found is necessary to meet.

Lyc. produces an unhealthy state of the milk—thickened milk; Cham., Phyto. have thickened, cheesy milk, mixed with pus. Lyc. also produces the opposite condition. The milk is thin and blue; child does not thrive because of the lack of nutrition. Acet-ac., Calc-c., Lach., Puls. are valuable remedies in this state and should be compared with Lyc. Women who never have milk for their infants. This is due to some constitutional wrong which you should endeavor to correct. Agnus-c., Asaf., Urtica-u., and Lac-can. are useful in such conditions.

These peculiarities of various remedies, suggested in the midst of the lecture, one by another, will perhaps become more strongly fixed in your memory for the coming times of need than if met in general reading.

Lyc. has great disturbance of the respiration; dyspnœa; difficult breathing; rousing up from sleep with great disturbance; fan-like motion of alæ nasi; oppression of breath, *agg.* by walking in open air; short breath in children, worse during sleep, worse during every exertion; asthmatic breathing. You will find "relief from fanning" in asthma, and that, being a symptom of the asthma and not of a drug, is not peculiar; it would be peculiar not to find it in that disease. Should you find "relief from fanning" in collapse, that would be peculiar, the symptom of a drug, and lead to CARB-V. or SULPH. as a remedy. You will hardly see asthma that does not desire to be fanned, to have more air. The Lyc. patient will sit at the window in winter weather and then wish to be fanned; must have the air in motion. A peculiar feature is the burning as of hot coals between the scapulæ; the patient will want the back fanned; there is the guide to Lyc.

Dry cough, day and night, with pains in the region of the stomach, and irritation of the respiratory tract as from the fumes of sulphur. The sputa is thick, milky, purulent, greenish yellow, with dark streaks of blood: this is the characteristic discharge. There is a dry cough in emaciated boys which is quite characteristic of Lyc. I have had several cases of boys who seemed about to go into a marasmus, in which Lyc. worked a perfect cure. The cough is *agg.* by eating; by drinking; by cold things (food and drink); by stooping; by lying down; by lying on the side—especially the left; in the wind, and in a warm room. Better by lying on the back; by sitting up. The Phos. and Puls. stomach symptoms are better from cold things, cold water, and *agg.* by warmth.

The so-called catarrh of the chest in its many variations often finds its simillimum in Lyc., especially after a neglected or badly treated pneumonia. Sulph. is also frequently indicated in this condition. Neglected pneumonia—perhaps after hepatization—

the symptoms appearing of abdominal breathing, heaving of the chest, little or no pain, more or less colliquative sweat, fan-like motion of the alæ, every sign of sinking, threatened paralysis of the lungs. The rattling in chest of Lyc. may be compared to Ant-t. Chest seems full of mucus.

Of the back we note chiefly the burning as of coals between the scapulæ. (Phos. has burning in a small spot in small of the back, better from rubbing; burning between the shoulders is found under Berb., Bry., Calc., Chelid., Ferr., Glon., Helon., Lyc., Mag-m., Merc., Nux-v., and Oxal-ac., Phos., Sulph.)

The pain in the sacral region of Lyc. is worse from rising from a seat; from stooping. Pain in lumbar region is *amel.* by passing urine.

Graph. has pain in the os coccygeus while urinating; Raph. has pain in lumbar region with urging to urinate; Caust. has a similar pain *after* copious flow of urine; Nat-sul. more closely resembles Lyc. in the symptom of pain in the back when retaining (too long) urine.

The rheumatic symptoms of this drug may be compared with those of Puls. and Rhus in the *amel.* from motion. Rhus leads, then Puls., next Lyc., especially in old cases. Tearing pains in shoulders and elbow joints during rest, not motion, is Lyc. Great dryness of palms of hands. Panaritium with gastric affections, think of Lyc. Gouty nodosities around joints; white swelling of the knees; coxalgia with violent jerks; child wakes cross or crying; profuse, fœtid foot sweat, with burning of the soles; one foot hot and the other cold; old ulcers on the legs with nightly stinging, burning, and itching.

Sleep of all kinds restless and uneasy, or deep and soporous. Child sleeps with eyes half open (Sulph.); throws its head from side to side; wakes cross, terrified, unrefreshed, and hungry.

The chill is from four to eight P. M.; the sweat is cold; chilly mornings the heat of the stove will not warm, and often as a concomitant we shall find red sand in the urine, sour vomiting, etc. In old, broken-down cases of malaria study Lyc.

There are many skin symptoms found under Lyc.; chiefly the skin is dry and hot; flesh in ridges, as if struck by a stick (Urticaria); blood boils. Such as do not mature but turn blue, (Lach.). In treating a patient who has ulcers we must not overlook this remedy, it is often indicated. Before finishing this lecture I must remind you that it is not well to begin the treatment of a chronic case with Lyc. It follows well after Sulph.,

Calc., or Lach.; and it will be followed well by Graph., Lach., Led., Phos., Sil. It is complementary to Iodine.

S. L. G. L.

ERRATA OF FIRST LECTURE OF LYCOPODIUM.

Page 462, line eleven, read *Tarent.*
Page 463, line eight, for *heat* read *heart.*
Page 468, line twelve, should read *Sulphate of Zinc.*

